# Synthesis and Biological Evaluation of 2-(3-Fluoro-4-nitro phenoxy)-*N*-phenylacetamide Derivatives as Novel Potential Affordable Antitubercular Agents

**DOI:** 10.3390/molecules17022248

**Published:** 2012-02-22

**Authors:** Wei Ang, Yan-Ni Lin, Tao Yang, Jian-Zhong Yang, Wei-Yi Pi, Ying-Hong Yang, You-Fu Luo, Yong Deng, Yu-Quan Wei

**Affiliations:** 1 Key Laboratory of Drug Targeting and Drug Delivery System of the Education Ministry, Department of Medicinal Chemistry, West China School of Pharmacy, Sichuan University, Chengdu 610041, Sichuan, China; 2 State Key Laboratory of Biotherapy, West China Hospital, West China Medical School, Sichuan University, Chengdu 610041, Sichuan, China; 3 Department of Pharmaceutical and Bioengineering, School of Chemical Engineering, Sichuan University, Chengdu 610065, Sichuan, China

**Keywords:** 2-(3-fluoro-4-nitrophenoxy)-*N*-phenylacetamide, antitubercular, H_37_Rv, rifampin-resistant, affordable

## Abstract

A novel series of 2-(3-fluoro-4-nitrophenoxy)-*N*-phenylacetamide compounds were designed, synthesized and *in vitro* assessed for their antitubercular activities by a microdilution method. All the novel derivatives exerted potent or moderate active against *M. tuberculosis* H_37_Rv, with MIC values ranging from 4 to 64 μg/mL. The most potent derivative **3m** showed an identical MIC value of 4 μg/mL for both *M. tuberculosis* H_37_Rv and rifampin-resistant *M. tuberculosis 261*. It demonstrated no inhibitory effects against six different tumor cell lines by a MTT assay and had a good safety profile in a vero cell line, providing a good lead for subsequent optimization in search of novel affordable antitubercular agents.

## 1. Introduction

Tuberculosis, or TB, is a common, and in many cases lethal, infectious disease caused by various strains of mycobacteria, usually *Mycobacterium tuberculosis*, which is the greatest single infection cause of higher mortality worldwide [1]. The World Health Organization (WHO) estimates that eight million people get TB every year and three million people will die yearly from TB if control is not further strengthened. The worsening situation has prompted the WHO to declare tuberculosis a global public health crisis [2,3]. The emergence of multidrug resistant (MDR) strains of *Mycobacterium tuberculosis* and the combined epidemics of HIV and tuberculosis, have worsened the situation. On the other hand, tuberculosis incidence rates correlate with poor socio-economic conditions. Consequently there is an urgent need to develop novel affordable antitubercular drugs with high potency that can provide treatment options for all forms of TB [4,5]. 

**Figure 1:**
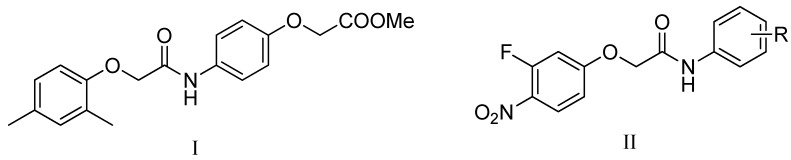
The chemical structures of methyl 2-(4-(2-(2,4-dimethylphenoxy)acetamido)phenoxy)acetate (**I**) and 2-(3-fluoro-4-nitrophenoxy)-*N*-phenylacetamide derivatives (**II**).

Compounds with a 2-phenoxy-*N*-phenylacetamide core structure have attracted considerable research interest as these entities have demonstrated a variety of biological activities such as anti-parasitic [6], anticancer [7] and antiviral effects [8]. Recently, in a program of high throughput screening for inhibitors of *M. tuberculosis* H_37_Rv, the hit compound methyl 2-(4-(2-(2,4-dimethylphenoxy)acetamido)phenoxy)acetate (**I**, Figure 1) [9], was identified to possess potent antitubercular activity, which indicates that 2-phenoxy-*N*-phenylacetamide may be a promising scaffold for develop novel antitubercular agents. The synthetic simplicity of the 2-phenoxy-*N*-phenylacetamide scaffold provides a strong motivation for the development of effective and affordable antitubercular agents. To date there have been no reports describing the synthesis and antitubercular assessment of its derivatives. Moreover, the antibacterial activity of halonitroanilides were documented and lots of compounds with nitro groups, such as nitrofurans and nitroimidazoles, have demonstrated broad spectrum antibacterial activities [10,11]. Encouraged by the prominent activity of the 2-phenoxy-*N*-phenylacetamide skeleton and the halonitroanilides moiety, we proposed to synthesize some novel compounds containing a 2-(3-fluoro-4-nitrophenoxy)-*N*-phenylacetamide nucleus by a molecular hybridization approach, aiming to search for potential candidates with better antitubercular activities and good safety profiles. Thus sixteen 2-(3-fluoro-4-nitrophenoxy)-*N*-phenylacetamide derivatives (**II**, Figure 1) were designed, synthesized and tested for their *in vitro* antitubercular activity against *M. tuberculosis* H_37_Rv by a microdilution method. The most potent one, compound **3m**, was assayed for its activities against two resistant *M. tuberculosis* strains isolated from clinical cases. The cytotoxic activities of all the final molecules against six tumor cell lines and normal vero cell line were assayed by MTT assay. 

## 2. Results and Discussion

### 2.1. Chemistry

To gain quick access to a range of final molecules for antitubercular activity evaluation, our initial synthetic effort was devoted to the synthesis of the pivotal intermediate, 2-(3-fluoro-4-nitrophenoxy)-acetic acid, by employing a routine Williamson ether reaction starting from 3-fluoro-4-nitrophenol and chloroacetic acid, which can be further condensed with an appropriate substituted aniline to afford the final compounds. Unfortunately, the simple Williamson reaction did not proceed to completion to afford the main product under the standard conditions for our specific substrates, because the fluorine atom *ortho* to the nitro group of 3-fluoro-4-nitrophenol is prone to hydrolysis under basic conditions. After laborious chromatographic workup, 2-(3-fluoro-4-nitrophenoxy)acetic acid can be obtained in very low yield. The following condensation step with several substituted anilines by dicyclohexylcarbodiimide (DCC) strategy did not proceed to completion and column chromatography must also be utilized to purify the target compounds.

**Scheme 1:**
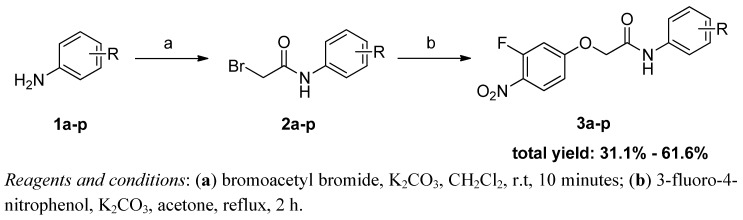
Synthetic route to2-(3-fluoro-4-nitrophenoxy)-*N*-phenylacetamide derivatives **3a–p**.

In order to make workup more convenient, we turned to a reported reaction sequence as shown in Scheme 1 [12]. In the first step, the intermediates **2a–p** were synthesized by treating the corresponding aromatic amines **1a–p** with bromoacetyl bromide at room temperature for 10 minutes in the presence of potassium carbonate. Then, without further purification of the crude products **2a–p**, the final compounds **3a–p** were obtained by refluxing certain **2a–p** in acetone with 3-fluoro-4-nitrophenol under basic conditions. Finally, all of the desired products **3a–p** were easily obtained by crystallization from ethyl acetate.

### 2.2. Antitubercular Activity

All the final compounds were evaluated *in vitro* as anti-tubercular agents by a microdilution method and their MIC values were shown in Table 1. 

**Table 1:** The MIC_90_ and MIC values of 2-(3-fluoro-4-nitrophenoxy)-*N*-phenylacetamide derivatives **3a–p** against *M. tuberculosis* H_37_Rv.

Compd.	R	MIC_90_^a^ (μg/mL)	MIC ^b^ (μg/mL)
**3a**	3-Cl	4	16
**3** **b**	3,4-Cl_2_	16	32
**3** **c**	4-Br,2-F	16	32
**3** **d**	4-Cl,3-CF_3_	8	16
**3** **e**	3-NO_2_	16	64
**3** **f**	2,4-(OCH_3_)_2_	16	64
**3** **g**	3,5-(CF_3_)_2_	4	8
**3** **h**	4-F,3-Cl	8	16
**3** **i**	4-Br	16	32
**3** **j**	2-CN	16	64
**3** **k**	2-CF_3_	32	64
**3** **l**	4-OCF_3_	16	32
**3** **m**	2-NO_2_	1	4
**3** **n**	4-Br,2-Cl	16	32
**3** **o**	4-CF_3_	8	16
**3** **p**	5-NO_2_,2-Me	64	64
**I**	-	1	4
**Isoniazide**	-	0.0625	0.0625

^a^ MIC_90_ = minimal drug concentration of the well in which the bacterial growth of the is similar to that with merely 100 CFU of bacteria inoculated; ^b^ MIC = minimal drug concentration of the well in which the bacterial growth is similar to that with merely 10 CFU of bacteria inoculated. Identical values were obtained for each compound in three replicates by visual investigation as stated in the experimental part.

To note, in our hands the MIC_90_ (1 μg/mL) or MIC (4 μg/mL) value of the reported hit compound **I** was less potent than stated. Compound **3m** with an *o*-nitroaniline moiety in the east part of the 2-phenoxy-*N*-phenylacetamide scaffold, showed an identical MIC value to the hit compound **I**, being the best active one among the novel 2-(3-fluoro-4-nitrophenoxy)-*N*-phenylacetamide derivatives with an MIC value of 4 μg/mL. However, compound **3e** and compound **3p** with a nitro group at the *meta*-position showed low inhibitory effects on H_37_Rv bacterial strain with an MIC value of 64 μg/mL. The underlying mechanism of this difference is not clear. The aniline bearing a trifluoromethyl group exhibited better activity (compound **3d** and compound **3o** with an identical MIC value of 16 μg/mL) except for *ortho*-trifluoromethyl (compound **3k** with an MIC values of 64 μg/mL). One chlorine atom in the aniline part (compound **3a** with an MIC value of 16 μg/mL) generated the slightly higher antitubercular activity relative to two chlorines (compound **3b** with an MIC value of 32 μg/mL) or one bromine atom substituent (compound **3i** with an MIC value of 32 μg/mL). Overall all the novel 2-(3-fluoro-4-nitrophenoxy)-*N*-phenylacetamide derivatives listed in Table 1 exerted potent or moderate active against *M. tuberculosis* H_37_Rv, which indicates that the 2-phenoxy-*N*-phenylacetamide scaffold is ideal for develop novel antitubercular agents by structural modification through various side chain introduction since our SARs information suggesting reasonable tolerance of functional groups changes.

Compound **3m** was further examined for its antitubercular activities against two resistant *M. tuberculosis* strains isolated from clinical cases and the results were reported in Table 2. As shown in Table 2, compound **3m** showed good activity against rifampicin-resistant TB with MIC of 4 μg/mL, and it also exhibited moderate activity against isoniazide-resistant TB with an MIC value of 32 μg/mL.

**Table 2:** The MIC values of compound **3m** against two resistant *M. tuberculosis* strains isolated from clinical cases ^a^.

Compd.	MIC ^b^ (μg/mL)	
	Isoniazide-resistant TB *242*	Rifampicin-resistant TB *261*
**3m**	32	4
**I**	16	4
**Isoniazide**	0.5	0.125
**Rifampicin**	≤0.25	>32

^a^ Clinical isolated single-drug resistant *M. tuberculosis* H_37_Rv were provided by Shanghai Pulmonary Hospital; ^b^ MIC = minimal drug concentration of the well in which bacterial growth is similar to that with only 10 CFU of bacteria inoculated. Identical values were obtained for each compound in three replicates by visual investigation as stated in the Experimental.

### 2.3. MTT-Based Cytotoxicity Assay

Compounds with MIC values ranging from 4 μg/mL to 16 μg/mL (compounds **3a**, **3d**, **3g**, **3h**, **3m** and **3o**) were chosen to conduct further cytotoxicity assays*.* The cytotoxic effects of these derivatives against six different cell lines were determined by the MTT assay method. The IC_50_ values of these compounds are listed in Table 3.

**Table 3:** The IC_50_ values of some 2-(3-fluoro-4-nitrophenoxy)-*N*-phenylacetamide derivativeson six tumor cell lines.

Compd.	IC_50_^a^ (μmol/L)						
	A375 ^b^	A549 ^c^	HepG2 ^d^	HCT116 ^e^	SKOV-3 ^f^	Hela ^g^	Vero ^h^
**3a**	>40.00	>40.00	>40.00	>40.00	>40.00	>40.00	>40.00
**3d**	>40.00	>40.00	>40.00	>40.00	>40.00	33.19 ± 2.5	>40.00
**3g**	22.61 ± 0.9	38.19 ± 1.5	21.64 ± 1.1	33.84 ± 2.0	12.18 ± 1.3	20.02 ± 1.1	>40.00
**3h**	>40.00	>40.00	>40.00	>40.00	>40.00	>40.00	>40.00
**3m**	>40.00	>40.00	>40.00	>40.00	>40.00	>40.00	>40.00
**3o**	28.29 ± 1.2	>40.00	>40.00	>40.00	28.60 ± 2.3	33.79 ± 1.7	>40.00

^a^ IC_50_ denotes half maximal inhibitory concentration. Values are means ± SEM of three independent experiments; ^b^ human melanoma cells; ^c^ lung adenocarcinoma epithelial cells; ^d^ hepatocellular liver carcinoma cells; ^e^ colorectal carcinoma cells; ^f^ ovarian carcinoma cells; ^g^ Henrietta Lacks strain cancer cells; ^h^ normal African green monkey kidney epithelial cells.

Most of the compounds showed low toxicity in this assay, and specifically the most potent compound **3m** exhibited a very good safety profile as its IC_50_ value was above 40 μmol/L against the tested six different cell lines.

## 3. Experimental

### 3.1. General

All solvents and reagents were analytical grade pure and used without further purification. All melting points were determined on a SGW X-4 Micro Melting Point apparatus and are uncorrected. ^1^H-NMR and ^13^C-NMR spectra were recorded on a Bruker Avance (Varian Unity Inova) 400 MHz spectrometer using TMS as internal reference chemical shift in δ, ppm. High-resolution mass spectrometry was carried out on a Waters Q-TOF Premier mass spectrometer.

### 3.2. Synthesis of Compounds ***3a–p***

Bromoacetyl bromide (1.18 eq.) was added dropwise to a mixture of the appropriate aniline **1a–p** (3.00 mmol, 1.0 eq.) and K_2_CO_3_ (1.18 eq.) in CH_2_Cl_2_ (10 mL) over 10 min. After cooling to room temperature, water (10 mL) was slowly added. The aqueous solution was extracted twice with CH_2_Cl_2_ (10 mL), the organic phase was dried over Na_2_SO_4_, filtered and the solvent was evaporated to furnish the crude product **2a–p**, which used in the next step without further purification (spectroscopic data were in agreement with published data). 

A mixture of the appropriate intermediate **2a–p** (1.0 eq.), 3-fluoro-4-nitrophenol (1.0 eq.), K_2_CO_3_ (1.2 eq.) and KI (0.1 eq.) in acetone (20 mL) was stirred at room temperature for 5 min and then refluxed for 2 h. The solvent was evaporated to give a residue which was suspended in H_2_O (20 mL) and extracted twice with EtOAc (20 mL). After drying over anhydrous Na_2_SO_4_, the organic layer was filtered and the solvent evaporated to dryness. The desired pure products **3a–p** were finally obtained by crystallization from EtOAc.

N*-(3-Chlorophenyl)-2-(3-fluoro-4-nitrophenoxy)acetamide* (**3a**): Pale yellow solid, total yield for the two steps: 48.7%; m.p. 150–151 °C; ^1^H-NMR (DMSO-*d_6_*): δ 10.39 (s, 1H), 8.19 (t, *J* = 1.6 Hz, 1H), 7.82 (s, 1H), 7.50 (d, *J* = 8.4 Hz, 1H), 7.37 (t, *J* = 8 Hz, 1H), 7.27 (d, *J* = 6.8 Hz, 1H), 7.16 (d, *J* = 7.6 Hz, 1H), 7.05 (d, *J* = 9.2 Hz, 1H), 4.95 (s, 2H); ^13^C-NMR (DMSO-*d_6_*): δ 165.98, 164.05, 158.05, 155.45, 139.95, 133.36, 130.80, 128.29, 123.79, 119.24, 111.94, 104.66, 67.68; HRMS (TOF) *m/z* calcd. for C_14_H_10_ClFN_2_O_4_ [M+Na^+^]: 324.0313, found: 347.0221.

N*-(3,4-Dichlorophenyl)-2-(3-fluoro-4-nitrophenoxy)acetamide* (**3b**): Yellow solid, total yield for the two steps: 61.6%; m.p. 175–176 °C; ^1^H-NMR (DMSO-*d_6_*): δ 10.49 (s, 1H), 8.19 (t, *J* = 9.2 Hz, 1H), 7.80 (d, *J* = 13.6 Hz, 1H), 7.62-7.53 (m, 2H), 7.27 (dd, *J* = 13.6 Hz, 2.4 Hz, 1H), 7.05(dd, *J* = 8.8 Hz, 2 Hz, 1H), 4.95 (s, 2H); ^13^C-NMR (DMSO-*d_6_*): δ 166.08, 163.93, 157.99, 155.39, 138.53, 131.25, 130.96, 128.24, 125.51, 121.02, 119.84, 104.73, 104.48, 67.61; HRMS (TOF) *m/z* calcd. for C_14_H_9_Cl_2_FN_2_O_4_ [M+Na^+^]: 382.9792, found: 382.9813.

N*-(4-Bromo-2-fluorophenyl)-2-(3-fluoro-4-nitrophenoxy)acetamide* (**3c**): Pale yellow solid, total yield for the two steps: 51.5%; m.p. 160–162 °C; ^1^H-NMR (DMSO-*d_6_*): δ 10.16 (s, 1H), 8.13 (t, *J* = 8.8 Hz, 1H), 7.83 (t, *J* = 8 Hz, 1H), 7.64 (d, *J* = 10 Hz, 1H), 7.41 (d, *J* = 8 Hz, 1H), 7.24 (d, *J* = 13.2 Hz, 1H), 7.03 (d, *J* = 8.8 Hz, 1H), 5.01 (s, 2H); ^13^C-NMR (DMSO-*d_6_*): δ 166.18, 164.11, 158.05, 155.34, 152.74, 128.27, 127.86, 125.93, 125.22, 119.29, 116.70, 111.91, 104.59, 67.61; HRMS (TOF) *m/z* calcd. for C_14_H_9_BrF_2_N_2_O_4_ [M+H^+^]: 386.9792, found: 387.1709.

N*-(4-Chloro-3-(trifluoromethyl)phenyl)-2-(3-fluoro-4-nitrophenoxy)acetamide* (**3d**): Pale yellow solid, total yield for the two steps: 58.9%; m.p. 163–164 °C; ^1^H-NMR (DMSO-*d_6_*): δ 10.58 (s, 1H), 8.21–8.16 (m, 2H), 7.90 (dd, *J* = 8.8 Hz, 2 Hz, 1H), 7.70 (d, *J* = 8.8 Hz, 1H), 7.27 (dd, *J* = 13.6 Hz, 2.4 Hz, 1H), 7.06 (dd, *J* = 9.2 Hz, 2 Hz, 1H), 4.97 (s, 2H); ^13^C-NMR (DMSO-*d_6_*): δ 166.36, 163.95, 158.05, 155.45, 137.99, 132.46, 128.26, 127.02, 124.72, 124.27, 121.55, 118.53, 111.98, 104.66, 67.65; HRMS (TOF) *m/z* calcd. for C_15_H_9_ClF_4_N_2_O_4_ [M+Na^+^]: 415.0085, found: 415.0099.

*2-(3-Fluoro-4-nitrophenoxy)-*N*-(3-nitrophenyl)acetamide* (**3e**): Yellow solid, total yield for two steps: 34.4%; m.p. 199–201 °C; ^1^H-NMR (DMSO-*d_6_*): δ 10.62 (s, 1H), 8.63 (s, 1H), 8.19 (t, *J* = 8.8 Hz, 1H), 8.00–7.95 (m, 2H), 7.65 (t, *J* = 8.4 Hz, 1H), 7.28 (dd, *J* = 13.6 Hz, 2.4 Hz,1H), 7.08 (dd, *J* = 9.2 Hz, 2 Hz, 1H), 4.99 (s, 2H); ^13^C-NMR (DMSO-*d_6_*): δ 166.42, 163.99, 158.05, 155.46, 148.18, 139.64, 130.68, 128.28, 125.86, 118.60, 113.99, 111.97, 104.68, 67.66; HRMS (TOF) *m/z* calcd. for C_14_H_10_FN_3_O_6_ [M+Na^+^]: 358.0451, found: 358.0467.

N*-(2,4-Dimethoxyphenyl)-2-(3-fluoro-4-nitrophenoxy)acetamide* (**3f**): Yellow solid, total yield for the two steps: 38.8%; m.p. 174–175 °C; ^1^H-NMR (DMSO-*d_6_*): δ 9.31 (s, 1H), 8.19 (t, *J* = 8.8 Hz, 1H), 7.71 (d, *J* = 8.8 Hz, 1H), 7.25 (dd, *J* = 9.6 Hz, 2.4 Hz, 1H), 7.04 (dd, *J* = 9.2 Hz, 1.6 Hz, 1H), 6.64 (d, *J* = 2.4 Hz, 1H), 6.50 (dd, *J* = 8.8 Hz, 2.8 Hz, 1H), 4.94 (s, 2H), 3.83 (s, 3H), 3.75 (s, 3H); ^13^C-NMR (DMSO-*d_6_*): δ 165.25, 163.99, 158.08, 157.47, 155.48, 151.76, 130.68, 128.29, 123.77, 119.58, 112.00, 104.50, 99.11, 67.89, 56.02, 55.56; HRMS (TOF) *m/z* calcd. for C_16_H_15_FN_2_O_6_ [M^+^]: 350.0914, found: 350.0787.

N*-(3,5-bis(Trifluoromethyl)phenyl)-2-(3-fluoro-4-nitrophenoxy)acetamide* (**3g**): Pale yellow solid, total yield for two steps: 32.8%; m.p. 168–169 °C; ^1^H-NMR (DMSO-*d_6_*): δ 10.82 (s, 1H), 8.32 (s, 2H), 8.20 (t, *J* = 9.2 Hz, 1H), 7.84 (s, 1H), 7.31 (dd, *J* = 13.6 Hz, 2.4 Hz, 1H), 7.09 (dd, *J* = 9.2 Hz, 2.4 Hz, 1H), 5.01 (s, 2H); ^13^C-NMR (DMSO-*d_6_*): δ 167.05, 164.03, 157.80, 156.07, 140.60, 131.25, 128.45, 126.31, 124.50, 122.70, 120.89, 119,83, 117.09, 112.23, 104.96, 104.80, 67.83; HRMS (TOF) *m/z* calcd. for C_16_H_9_F_7_N_2_O_4_ [M+Na^+^]: 449.0348, found: 449.0328.

N*-(3-Chloro-4-fluorophenyl)-2-(3-fluoro-4-nitrophenoxy)acetamide* (**3h**): Yellow solid, total yield for two steps: 56.8%; m.p. 166–167 °C; ^1^H-NMR (DMSO-*d_6_*): δ 10.40 (s, 1H), 8.20 (t, *J* = 8.8 Hz, 1H), 7.93 (dd, *J* = 6.8 Hz, 2.4 Hz, 1H), 7.55–7.51 (m, 1H), 7.41 (t, *J* = 9.2 Hz, 1H), 7.27 (dd, *J* = 13.6 Hz, 2.4 Hz, 1H), 7.06 (dd, *J* = 9.2 Hz, 2 Hz, 1H), 4.94 (s, 2H); ^13^C-NMR (DMSO-*d_6_*): δ 166.08, 164.17, 157.80, 156.06, 154.68, 153.07, 135.90, 130.96, 128.45, 121.60, 120.47, 119.64, 117.45, 112.16, 104.86, 67.88; HRMS (TOF) *m/z* calcd. for C_14_H_9_ClF_2_N_2_O_4_ [M+Na^+^]: 365.0117, found: 365.0143.

N*-(4-Bromophenyl)-2-(3-fluoro-4-nitrophenoxy)acetamide* (**3i**): Pale yellow solid, total yield: 42.2%; m.p. 166–167 °C; ^1^H-NMR (DMSO-*d_6_*): δ 10.33 (s, 1H), 8.19 (t, *J* = 8.8 Hz, 1H), 7.59 (d, *J* = 8.8 Hz, 1H), 7.52 (d, *J* = 8.8 Hz, 2H), 7.26 (dd, *J* = 13.2 Hz, 1.6 Hz, 1H), 7.05 (dd, *J* = 9.2 Hz, 2.4 Hz, 1H), 4.94 (s, 2H); ^13^C-NMR (DMSO-*d_6_*): δ 165.91, 164.28, 157.81, 156.07, 138.08, 132.08, 130.93, 128.45, 122.00, 115.92, 112.12, 104.84, 67.97; HRMS (TOF) *m/z* calcd. for C_14_H_10_BrFN_2_O_4_ [M+Na^+^]: 392.9685, found: 392.9710.

N*-(2-Cyanophenyl)-2-(3-fluoro-4-nitrophenoxy)acetamide* (**3j**): White solid, total yield for the two steps: 31.1%; m.p. 172–174 °C; ^1^H-NMR (DMSO-*d_6_*): δ 10.45 (s, 1H), 8.20 (t, *J* = 9.2 Hz, 1H), 7.86 (d, *J* = 7.6 Hz, 1H), 7.52 (d, *J* = 8.8 Hz, 2H), 7.73 (t, *J* = 8 Hz, 1H), 7.65 (d, *J* = 8 Hz, 1H), 7.41 (t, *J* = 6.8 Hz, 1H), 7.27 (dd, *J* = 13.6 Hz, 2 Hz, 1H), 7.07 (d, *J* = 9.2 Hz, 1H), 5.03 (s, 2H); ^13^C-NMR (DMSO-*d_6_*): δ 166.63, 164.02, 157.81, 156.08, 139.70, 134.41, 133.74, 131.04, 128.43, 126.73, 126.15, 117.14, 112.36, 108.13, 104.87, 67.88; HRMS (TOF) *m/z* calcd. for C_15_H_10_FN_3_O_4_ [M+Na^+^]: 338.0553, found: 338.0544.

*2-(3-Fluoro-4-nitrophenoxy)-*N*-(2-(trifluoromethyl)phenyl)acetamide* (**3k**): colorless crystal, total yield for the two steps: 39.7%; m.p. 146–147 °C; ^1^H-NMR (DMSO-*d_6_*): δ 9.94 (s, 1H), 8.20 (t, *J* = 9.2 Hz, 1H), 7.78 (d, *J* = 6.8 Hz, 1H), 7.72 (t, *J* = 8 Hz, 1H), 7.57 (d, *J* = 8 Hz, 1H), 7.51 (t, *J* =7.6 Hz, 1H), 7.22 (dd, *J* = 13.2 Hz, 2.4 Hz, 1H), 7.04 (dd, *J* = 9.2 Hz, 2 Hz, 1H), 4.98 (s, 2H); ^13^C-NMR (DMSO-*d_6_*): δ 166.99, 163.99, 157.79, 156.06, 134.89, 133.66, 131.00, 128.44, 127.67, 126.89, 124.86, 123.05, 112.31, 104.71, 67.85; HRMS (TOF) *m/z* calcd. for C_15_H_10_F_4_N_2_O_4_ [M+Na^+^]: 381.0474, found: 381.0459.

*2-(3-Fluoro-4-nitrophenoxy)-*N*-(4-(trifluoromethoxy)phenyl)acetamide* (**3l**): Grey white solid, total yield for the two steps: 40.0%; m.p. 146–148 °C; ^1^H-NMR (DMSO-*d_6_*): δ 10.40 (s, 1H), 8.19 (t, *J* = 9.2 Hz, 1H), 7.74–7.71 (m, 2H), 7.35 (d, *J* = 8.4 Hz, 2H), 7.26 (dd, *J* = 13.6 Hz, 2.8 Hz, 1H), 7.05 (dd, *J* = 9.2 Hz, 2 Hz, 1H), 4.95 (s, 2H); ^13^C-NMR (DMSO-*d_6_*): δ 165.99, 164.28, 155.65, 144.42, 137.90, 130.96, 128.45, 121.85, 119.31, 112.14, 104.85, 67.96; HRMS (TOF) *m/z* calcd. for C_15_H_10_F_4_N_2_O_5_ [M+K^+^]: 397.0214, found: 397.0431.

*2-(3-Fluoro-4-nitrophenoxy)-*N*-(2-nitrophenyl)acetamide* (**3m**): Yellow solid, total yield for the two steps: 39.2%; m.p. 201–202 °C; ^1^H-NMR (DMSO-*d_6_*): δ 10.77 (s, 1H), 8.22 (t, *J* = 9.2 Hz, 1H), 8.07 (dd, *J* = 8 Hz, 1.2 Hz, 1H), 7.95 (d, *J* = 8 Hz, 1H), 7.79–7.75 (m, 1H), 7.44-7.39 (m, 1H), 7.27 (dd, *J* = 13.6 Hz, 2.8 Hz, 1H), 7.08 (dd, *J* = 9.2 Hz, 2.4 Hz, 1H), 5.00 (s, 2H); ^13^C-NMR (DMSO-*d_6_*): δ 166.47, 163.60, 158.25, 155.65, 141.48, 135.16, 131.25, 128.51, 125.84, 125.09, 122.48, 104.85, 67.88; HRMS (TOF) *m/z* calcd. for C_14_H_10_FN_3_O_6_ [M+Na^+^]: 358.0451, found: 358.0452.

N*-(4-Bromo-2-chlorophenyl)-2-(3-fluoro-4-nitrophenoxy)acetamide* (**3n**): White solid, total yield for two steps: 54.1%; m.p. 173–175 °C; ^1^H-NMR (DMSO-*d_6_*): δ 9.90 (s, 1H), 8.20 (t, *J* = 9.2 Hz, 1H), 7.83 (d, *J* = 2 Hz, 1H), 7.73 (d, *J* = 8.8 Hz, 1H), 7.58 (dd, *J* = 8.4 Hz, 2 Hz, 1H), 7.27 (dd, *J* = 9.2 Hz, 2.8 Hz, 1H), 7.06 (dd, *J* = 9.2 Hz, 2 Hz, 1H), 5.02 (s, 2H); ^13^C-NMR (DMSO-*d_6_*): δ 166.37, 164.04, 158.24, 155.64, 134.12, 132.18, 131.06, 128.39, 127.76, 118.25, 112.23, 104.89, 67.95; HRMS (TOF) *m/z* calcd. for C_14_H_9_BrClFN_2_O_4_ [M+Na^+^]: 426.9295, found:426.9032.

*2-(3-Fluoro-4-nitrophenoxy)-*N*-(4-(trifluoromethyl)phenyl)acetamide* (**3o**): Pale yellow solid, total yield for the two steps: 36.1%; m.p. 173–175 °C; ^1^H-NMR (DMSO-*d_6_*): δ 10.57 (s, 1H), 8.19 (t, *J* = 9.6 Hz, 1H), 7.84 (d, *J* = 8.8 Hz, 2H), 7.72 (d, *J* = 8.4 Hz, 2H), 7.28 (dd, *J* = 13.6 Hz, 2.4 Hz, 1H), 7.06 (dd, *J* = 9.2 Hz, 2 Hz, 1H), 4.99 (s, 2H); ^13^C-NMR (DMSO-*d_6_*): δ 166.42, 164.27, 158.25, 155.65, 142.31, 130.96, 128.45, 126.54, 126.10, 124.28, 123.40, 119.96, 112.13, 104.85, 67.95; HRMS (TOF) *m/z* calcd. for C_15_H_10_F_4_N_2_O_4_ [M+Na^+^]: 381.0474, found: 381.0473.

*2-(3-Fluoro-4-nitrophenoxy)-*N*-(2-methyl-5-nitrophenyl)acetamide* (**3p**): Pale yellow solid, total yield for two steps: 36.9%; m.p. 211–213 °C; ^1^H-NMR (DMSO-*d_6_*): δ 9.90 (s, 1H), 8.44 (d, *J* = 2.4 Hz, 1H), 8.21 (t, *J* = 9.2 Hz, 1H), 7.99 (dd, *J* = 8.4 Hz, 2.4 Hz, 1H), 7.54 (d, *J* = 8.8 Hz, 1H), 7.29 (dd, *J* = 13.6 Hz, 2.4 Hz, 1H), 7.08 (dd, *J* = 9.2 Hz, 2 Hz, 1H), 5.04 (s, 2H), 2.36 (s, 3H); ^13^C-NMR (DMSO-*d_6_*): δ 166.57, 164.24, 158.27, 155.67, 146.18, 140.07, 136.88, 131.96, 130.99, 128.48, 120.36, 119.26, 112.19, 104.86, 67.90; HRMS (TOF) *m/z* calcd. for C_15_H_12_FN_3_O_6_ [M+Na^+^]: 372.0608, found: 372.0599.

### 3.3. Antitubercular Activity

Middlebrook7H9 medium dry powder and nutrition supplements (OADC) were bought from the Becton Dickinson (BD) Company (Franklin Lakes, NJ, USA). The *M. tuberculosis* H_37_Rv (ATCC 27294) were provided by national strains preservation center. Clinical isolated single-drug resistant *M. tuberculosis* H_37_Rv were provided by the Shanghai Pulmonary Hospital.

Organisms for testing were initially grown to turbidity in 7H9 broth for 14 days. The cultures were vortexed for 20 to 30 seconds before use and then left for 10 to 30 minutes to allow for settling of heavy particles. The suspension of organisms was then matched to the optical density of a 1.0 McFarland standard. Appropriate dilutions were added to the wells of the microtiter plates containing the different drug dilutions by using a disposable inoculator. The tested compounds were added to the medium as DMSO solutions. The following concentrations were used: 64, 32, 16, 8, 4, 2, 1, 0.5, 0.25, 0.125, 0.0625, 0.03125 μg/mL. The plates were sealed in plastic bags and incubated at 37 °C in room air in a moisturized incubator. Isoniazide (Sigma-Aldrich, St. Louis, MO, USA) was used as positive control in each experiment, and each evaluation was performed in triplicate. The plastic bags were essential to prevent evaporation in the wells and were also useful for biologic containment. All microdilution plates were read after 11 days by visual investigation with an indirect light source.

### 3.4. MTT-Based Cytotoxicity Assay

Briefly, cells (2,500/well) were seeded in 96-well plates and cultured for 24 h, followed by treatment with the target compounds for another 48 h. MTT (5 μg/mL, 20 μL) was added per well and incubated for another 2.5 h at 37 °C. Then the supernatant fluid was removed and 150 μL/well DMSO was added for 15–20 min. The absorbance (OD) of each well was measured at 570 nm, using a SpectraMAX M5 microplate spectrophotometer (Molecular Devices, Sunnyvale, CA, USA). The IC_50_ Values are means ± SEM of three independent experiments.

## 4. Conclusions

Sixteen 2-(3-fluoro-4-nitrophenoxy)-*N*-phenylacetamide derivatives were synthesized and their antimycobacterial activities against *M. tuberculosis* H_37_Rv evaluated by a microdilution method. The newly synthesized derivatives have high to moderate potency against *M. tuberculosis* H_37_Rv, which indicates that the readily available 2-phenoxy-*N*-phenylacetamide scaffold is ideal for developing novel affordable antitubercular agents by structural modification. Six compounds exhibited potent antitubercular activity, with MIC values ranging from 4 to 16 μg/mL, and 2-(3-fluoro-4-nitrophenoxy)-*N*-(2-nitrophenyl)acetamide (compound **3m**) was proved to be the most potent inhibitor with an identical MIC value of 4 μg/mL for *M. tuberculosis* H_37_Rv and rifampin-resistant *M. tuberculosis* 261 strain isolated from clinical cases. It also has a good safety profile in six different cell lines by the MTT assay, indicating compound **3m** be a good lead for subsequent optimization to get better antitubercular agents.
